# Relationship between disease perception and feelings of powerlessness in lymphoma patients: the mediating effect of social support and level of hope

**DOI:** 10.3389/fpsyt.2025.1557867

**Published:** 2025-02-19

**Authors:** Yingying Zhu, Haiying Hua, Li Sheng, Jingfen Zhou, Long Ye, Siyu Gu

**Affiliations:** ^1^ Graduate School of Wuxi Medical College, Jiangnan University, Wuxi, China; ^2^ Department of Hematology, Affiliated Hospital of Jiangnan University, Wuxi, China; ^3^ Nursing Department, The Second Affiliated Hospital of Zhejiang University School of Medicine, Hangzhou, China

**Keywords:** lymphoma, disease perception, feeling of powerlessness, social support, level of hope, mediated effects

## Abstract

**Objectives:**

Lymphoma patients often experience significant physical and psychological stress, with feelings of powerlessness negatively impacting their health. While social support and hope are crucial for improving mood and coping with disease, their mechanisms in relation to disease perception and powerlessness remain unclear. This study aimed to examine the relationship between disease perception and feelings of powerlessness in lymphoma patients, focusing on the mediating roles of social support and hope.

**Methods:**

For a cross-sectional design, 311 lymphoma patients were surveyed using the Brief illness perception questionnaire, Powerlessness assessment tool, Social Support Rating Scale, and Herth’s Hope Scale, and were statistically analyzed using the software SPSS 27.0 and PROCESS version 4.1.

**Results:**

A correlation was found between the disease perception, social support, hope level, and feeling of powerlessness of lymphoma patients (*P* < 0.01). There was a positive correlation between perceptions of illness and powerlessness (β= 0.291, *P*<0.001), and disease perception could influence powerlessness through three indirect pathways: the separate mediating effect of social support [β= 0.057, 95%CI (0.029~0.091)], the separate mediating effect of level of hope [β= 0.07, 95%CI (0.034~ 0.107)], and the chain mediating effect of social support and hope level [β= 0.019, 95%CI (0.008~0.033)]. Social support and level of hope played a partial medium mediating effect in the effect of perceived disease on feelings of powerlessness, accounting for 13.04% and 16.02% of the total effect, respectively, and the chained mediating effect of both accounted for 4.35% of the total effect.

**Conclusions:**

Disease perception and powerlessness were positively correlated in lymphoma patients, and in addition, social support and level of hope mediated the relationship. This conclusion provides a theoretical basis and guidance for nursing interventions to reduce powerlessness in lymphoma patients.

## 1 Introduction

Lymphomas are a highly heterogeneous group of malignant tumors that originate in the hematopoietic system and are characterized by the abnormal proliferation of mature lymphocytes or their precursors (1). Lymphoma is a common cause of death, with approximately 590,000 new cases worldwide (2), and its annual incidence is increasing (3). In China, the incidence of lymphoma is increasing by approximately 5% per year, making it one of the top ten most prevalent tumors in the country (4, 5). Currently, malignant lymphoma is the most common type of hematological tumor, and its pathological classifications include Hodgkin’s and non-Hodgkin’s lymphomas (6). Lymphoma patients often face a burden of systemic and localized symptoms (7). Compared to other solid tumors, lymphoma has a complex pathological type, long treatment cycles and a tendency for the disease to progress or recur (8). The process of moving from symptoms to diagnosis and treatment can be distressing for many patients (9, 10). Disease perception is an individual’s view of the disease and influences the patient’s behavior (11). And Garba et al. (12) showed that patients’ negative perceptions are a risk factor for the severity of their symptoms, and that patients’ loss of control over the perception of their disease symptoms affects their physical and mental health and their level of quality of life (12, 13). The negative perceptions of patients are a risk factor for the severity of their symptoms. The experience of lymphoma, from symptoms to diagnosis and treatment, can be painful for many patients (9, 10). Lymphoma is mainly treated with radiotherapy and chemotherapy, which not only brings a huge symptomatic burden to patients but also imposes a heavy financial burden, resulting in serious psychological problems (14). Psychological distress is a common problem in patients (15). Among these, anxiety and depression are the most common negative emotions (16), which may be accompanied by emotional reactions such as nervousness, fear, low mood, and pessimism. Among these, the feeling of powerlessness (FOP) is closely related to serious illness and suffering and is regarded as one of the criteria for depression in end-stage patients (17). Prolonged feelings of powerlessness may lead to a decrease in self-esteem, an increase in despair, and even a tendency toward self-harm, ultimately resulting in a significant decrease in quality of life (18). The harm caused by powerlessness has been gradually discovered by scholars, but no study has focused on patients with lymphoma. In addition, most previous studies have focused on exploring the relationship between feelings of powerlessness and single variables such as social support (19, 20), quality of life (21), and self-efficacy (22); however, no study has yet explored the relationship between disease perception and powerlessness in patients with lymphoma, and the level of patients’ perceived disease control is closely related to their mood (23), treatment adherence (24, 25) and prognosis (26), which is one of the predictors of patients’ health outcomes (27). Whereas exploring the relationship between them is important for understanding the psychological state of patients with lymphoma and for improving the outcome of their treatment, the present study aimed to explore the relationship between disease perceptions and powerlessness in patients with lymphoma, and given the importance of positive psychology and social determinants, this study combined levels of hope and social support (including family support); to explore their mediating role between disease perceptions and powerlessness, with the aim of elucidating the potential mechanisms through which these factors influence patient outcomes.

## 2 Theoretical framework and literature review

### 2.1 Disease perception and FOP

Disease perception, also known as disease cognition, is the process by which an individual analyzes, interprets, and develops an understanding and emotional response to a symptom or disease based on personal experience in the face of a health threat (28), which typically includes the identity of the disease (name and symptom), etiology, duration, personal impact, and sense of control (29). Disease perception is a key aspect of how individuals perceive and respond to their health conditions. Positive perceptions can improve quality of life and reduce symptom intensity, whereas negative perceptions can lead to poor health and increased emotional distress (30), which in turn affects disease prognosis and return-to-work rates (31). Previous studies (8) have confirmed that negative disease perceptions are at high levels in patients with lymphoma, and Newcomb et al. (32) found that patients with lymphoma suffer from severe psychological distress and have a complex cognitive understanding of their prognosis. Segal et al. (33) explored the role of disease perceptions in patients with lymphoma, emphasizing the importance of understanding and addressing disease perceptions within the context of lymphoma care. The above literature demonstrates the impact of disease perceptions on the experiences and outcomes of patients with lymphoma; therefore, understanding and improving the perceptions of patients with lymphoma about their disease is critical to providing comprehensive care and improving overall health outcomes.

The FOP is a psychosocial phenomenon typically triggered by health issues or significant life events (34). Research has indicated that FOP is a critical risk factor for poor physical and mental health (35). Individuals with chronic illnesses frequently experience uncertainty regarding their health status during recovery or as their physiological functioning gradually declines. Furthermore, the unpredictable nature of their condition contributes to a heightened FOP (21). Consequently, this FOP is not only a significant psychological concern that warrants attention but also directly influences patients’ abilities and responses when confronting their illness and undergoing treatment (36). FOPs often infiltrate patients’ minds after unsuccessful attempts to combat the disease, rendering them emotionally vulnerable. This emotional state adversely affects patients, leaving them feeling weak, powerless, and unable to exert control over their lives, whether mentally, physically, or financially (37).

The relationship between disease perception and FOP in lymphoma patients and its underlying mechanisms remain unknown. Previous studies (38) have found that disease perception is associated with anxiety and depression, which are risk factors for disease perception. Feelings of powerlessness are also associated with anxiety and depression (17, 39) as depressed individuals often experience a lack of control and hope. Powerlessness (40) reduces feelings of personal control, which in turn reduces self-centered counterfactual thinking. This suggests that FOP may be related to the cognitive component of disease perception, specifically feelings of personal control. Disease perception encompasses both cognitive and emotional aspects. Given this, emotional responses and perceived personal control within this framework are hypothesized to have a direct influence on FOP.

### 2.2 The mediating role of social support

In the face of stress, people often produce a variety of negative emotions, which in turn negatively affect their physiological health. Care or help from family members or other social relationships protects the healthy development of human psychology and physiology. Patients’ social support is negatively correlated with their perception of disease (41) and can directly affect their FOP (42–44). Another investigation (45) showed that patients lacking social support often lacked someone to confide in when facing the stress caused by their illness and therefore showed low confidence in their treatment, which made them more prone to FOP. In addition, family and social support have a more significant impact on patients’ FOP than factors related to the disease itself (44). This finding may suggest that social support plays a mediating role in the relationship between patients’ perceptions of illness and FOP.

### 2.3 The mediating role of the level of hope

With the rise of positive psychology and its widespread application in various fields, hope has gradually become an important part of research. Studies have shown that hope can help cancer patients cope with their disease more effectively and improve their positive health behaviors, thus reducing depression to a certain extent (46, 47). In addition, other studies found that a patient’s level of illness perception had a significant impact on the level of hope (48, 49). Hope plays an important mediating role in illness perception and quality of life (50). Hope has been shown to be (51) negatively associated with symptom burden, psychological distress, and depression, whereas FOP can be moderated by hope (39). There is also literature showing a complex relationship between hope and powerlessness, and that hope is often a source of strength and motivation in the face of powerlessness (52). Therefore, the level of hope may be one of the mediating variables between the perception of disease and the FOP in lymphoma patients.

### 2.4 The chain mediation of social support and level of hope

This study adopted the Common Sense Model of Self-Regulation (53)(CSM) as a theoretical framework, which focuses on the interrelationships between individuals’ illness perceptions, coping strategies, and health outcomes after they develop an illness. The CSM emphasizes how individuals assess health threats and adopt coping strategies based on their perceptions of illness and how these perceptions and behaviors affect their health outcomes (54). In lymphoma patients, CSM can be used to explore how illness perceptions influence emotional reactions and coping strategies and how these factors further influence FOP and social support-seeking behaviors (55). In addition, CSM emphasizes the role of social support and hope levels as mediating variables, revealing how they influence the relationship between illness perception and FOP (55). Previous studies have found that social support has a significant influence on hope levels and that patients with higher levels of social support have higher hope levels (56–58). This suggests that hope levels and social support can regulate patients’ psychological stress, increase confidence in disease recovery, and alleviate feelings of helplessness, thus reducing their FOP. Social support not only affects helplessness through a single mediator variable but may also work simultaneously through multiple mediator variables (59). This suggests that social support may indirectly affect FOP by influencing individuals’ levels of hope.

In summary, We aimed to explore the relationship between illness perception and FOP in lymphoma patients, and investigate the mediating role of social support and levels of hope.

**Figure 1:**
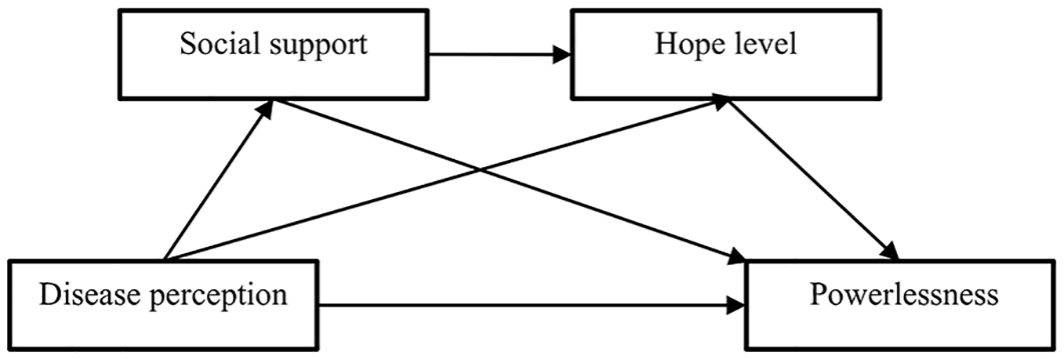
Hypothetical model of chain mediation in lymphoma patients.

Therefore, we formulated the following hypotheses:

Hypothesis 1: Illness perception and FOP were positively correlated.
Hypothesis 2: Social support mediates the relationship between illness perception and FOP.
Hypothesis 3: Hope level mediates the relationship between illness perception and FOP.
Hypothesis 4: Social support and levels of hope may modulate the relationship between illness perception and FOP. (The hypothesized model is illustrated in **Figure 1**).

## 3 Research methodology

### 3.1 Study design

We employed a cross-sectional design focusing on patients with lymphoma who received inpatient treatment at the Affiliated Hospital of Jiangnan University between November 2023 and October 2024.

### 3.2 Participants

Participants were recruited using a convenience sampling method with the following eligibility criteria: (1) patients had been diagnosed with lymphoma diagnosis according to the Lymphoma Diagnostic and Treatment Guidelines (2022 Edition) (60); (2) age between 18 and 90 years; (3) Participants are required to have basic Chinese comprehension and expression skills, with the ability to accurately understand the questions and respond clearly; (4) clear comprehension of condition and ability to cooperate in completing the survey in a stable condition; and (5) ability to provide written informed consent and participate voluntarily.

The exclusion criteria were as follows: (1) patients with a history of mental illness or a current state of mental disorder, (2) severe cognitive impairment or difficulty comprehending the questionnaire, (3) serious life-threatening conditions at any time, and (4) patients with other serious medical conditions that could interfere with the results of the study or with the ability to participate.

### 3.3 Measurement tools

#### 3.3.1 General information questionnaire

The design of this study was determined based on the researcher’s extensive review of the literature and in-depth discussions with the members of the subject group. It covered two main sections: general demographic information and disease-related information. The general demographic data included information on participants’ gender, age, education level, marital status, mode of residence, type of health insurance, sleep, and social activities; on the other hand, the disease-related data recorded detailed information on patients’ self-care ability, complications, disease recurrence, the specific type of the disease, the duration of the disease, and the treatment modalities adopted Information.

#### 3.3.2 Illness perception

In this study, illness perception was assessed using the Brief Illness Perception Questionnaire (BIPQ) (61), The questionnaire consists of nine items, but item 9 is an open-ended question, so it is not scored. The total score ranges from 0 to 80, with higher scores indicating higher levels of negative illness perception in individuals. The scale has been widely used to measure illness perception in various populations. The questionnaire has good psychometric properties, and Sun Weiming et al. (62) Chineseized it in 2015 and measured a Cronbach’s α coefficient of 0.831, which has good reliability and validity, and measured a Cronbach’s α coefficient of 0.797 in this study, which indicates that the scale is suitable for use in this study.

#### 3.3.3 Feelings of powerlessness

To assess the FOP, the Chinese version of the Perceived Adult Powerlessness Scale (PAT) was used, as described by Huang Yao et al. (63) in 2018. The overall Cronbach’s alpha coefficient was verified to be 0.96. There are twelve entries in the PAT, including 2 dimensions, which are the self-perceived dimensions of executive behaviors and decision-making abilities, and the emotional responses of perceived self-control. The scores range from 12 to 60 on a five-point scale: 0-12 none, 12-24 mild, 25-36 moderate, 37-48 severe, and 49-60 extremely severe, with high scores indicating a strong FOP. In this study, the Cronbach’s alpha coefficient of the PAT was 0.887, indicating good reliability and validity.

#### 3.3.4 Social support

The Social Support Rating Scale (SSRS) (64) was used, which is divided into three dimensions of objective support, subjective support, and social support utilization, with 10 items, and its Cronbach’s alpha coefficient is 0.896. The total score of this scale ranges from 12 to 66 points, with 12 to 22 points as low level, 23 to 44 points as medium level, and 45 to 66 points as high level. The higher the score, the higher the level of social support. The Cronbach’s alpha coefficient of this scale in this study is 0.745, which indicates good reliability.

#### 3.3.5 Level of hope

Patient hope levels were assessed using the Herth Hope Inventory (HHI) (65), which has 12 entries in total, and the scale has good internal consistency reliability after Chineseization, with Cronbach’s alpha coefficient of 0.85. The total score of the scale is 12~48, with 12~23 representing a low level of hope, 24~35 representing a medium level of hope, and 36~48 representing a high level of hope, and the higher the total score, the higher the hope level of the patient. The higher the total score, the higher the level of hope of the patient. HHI is one of the most commonly used tools to evaluate the level of hope. The Cronbach’s alpha coefficient in this study was 0.868, which has good reliability and validity.

### 3.4 Data collection

This study was approved by the Medical Ethics Committee of the Jiangnan University Hospital (No. Ls2024034). Before conducting the survey, we obtained consent from the director and head nurse of the department in which we worked. During the survey period, two systematically trained researchers visited the wards to collect data while avoiding disturbance to the patients’ treatment and rest time. After obtaining the patients’ verbal or written informed consent, a self-reported paper questionnaire was provided to them; the researchers patiently replied to any questions from the patients and guided them to complete the questionnaire. After completion, the researchers collected the questionnaires uniformly and checked for errors. Before data collection, anonymity was emphasized to ensure accuracy and completeness. A total of 325 questionnaires were distributed and 311 valid questionnaires were returned, with a valid recovery rate of 95.69%.

### 3.5 Quality control

In this cross-sectional study, various control measures were implemented to effectively address potential biases and confounding variables. To obtain a well-representative sample and minimize selection bias, random sampling techniques were utilized to ensure that participants met the recruitment criteria. During the data collection phase, strict adherence to standardized operating procedures was maintained, which included the use of validated instruments and comprehensive training for data collectors to ensure consistency in data collection. Furthermore, appropriate strategies for handling missing data, such as multiple imputation, were adopted to mitigate the risk of bias. Ultimately, these strategies significantly reduce potential biases and enhance the reliability of the research findings.

### 3.6 Data analysis methods

Epidata software was used for two-person data entry. Data analysis was conducted using SPSS version 27.0 along with its Process 4.1 plug-in. Descriptive statistics were reported such that measurement data conforming to a normal distribution are expressed as mean ± standard deviation (x ± s), while skewed data are presented as median and interquartile range [m (P25, P75)]. Count data were analyzed using frequency and composition ratios. To investigate the correlation between variables, Pearson correlation analysis was utilized. Additionally, the Bootstrap method was applied to conduct a detailed analysis of the mediating effects. Model 6 was chosen to assess the mediating effects of social support and hope level, with a significance threshold set at *P* < 0.05.

## 4 Results

### 4.1 Common method deviation test

Harman’s one-factor test (66) results showed that 10 factors had eigenvalues exceeding 1, with the first factor explaining 27.0% of the variance. This result was below the critical criterion of 40%, indicating that no significant common method bias existed in the data used in this study.

### 4.2 Characteristics of general demographic and disease-related information

**Table 1** shows the demographics and characteristics of the participants; 311 patients were included in the final analyses. The minimum patient age was 18 years. The maximum age was 89 years, with a mean age of 63.48 years (SD 13.34), and 89.4% of the patients had an education level of high school/secondary school or below. Regarding marital status, 91.3% of patients had spouses. In terms of disease stage, stages III-IV accounted for more than 70% of cases, and in terms of treatment modality, 67.2% of patients were treated with chemotherapy, 24.8% with maintenance therapy, and 8% with follow-up status. Regarding the type of lymphoma, Hodgkin’s lymphoma accounted for 5.5% and non-Hodgkin’s lymphoma accounted for 94.5%. In terms of disease duration, the time of diagnosis was less than 3 months in 34.4% of cases, and the disease was more than 2 years in 25.7% of cases.

**Table 1:** General demographic and disease-related information characterizing patients with lymphoma (n = 311).

Variable	Subgroup	N	Composition ratio (%)
Sex	Male	165	53.1
	Female	146	46.9
Age	≤ 45 years old	34	10.9
	46-60 years	76	24.4
	61-75 years	147	47.3
	≥ 76 years old	54	17.4
Educational level	Elementary or middle school	226	72.7
	High school or junior college	52	16.7
	College and above	33	10.6
Marital status	With spouse	284	91.3
	No spouse	27	8.7
	Passive	173	55.6
Type of medical insurance	Self-financed	21	6.8
	Urban residents' medical insurance	86	27.7
	Urban workers' medical insurance	204	65.6
Sleep	Enough	104	33.4
	General	144	46.3
	Insufficient	63	20.3
Ecog score	0-2 points	221	71.1
	3 points	69	22.2
	4 points	21	6.8
Socialization	Participation	146	46.9
	Non-participation	165	53.1
Complication	Yes	126	40.5
	None	185	59.5
Pain	Yes	85	27.3
	None	226	72.7
Type of disease	Hodgkin's lymphoma	17	5.5
	Non-hodgkin lymphoma	294	94.5
Disease staging	I~II	88	28.3
	III~IV	223	71.7
Duration of disease	≤ 3 months	107	34.4
	3-6 months	41	13.2
	6 months-2 years	83	26.7
	> 2 years	80	25.7
Treatment	Initial chemotherapy	172	55.3
	Relapse chemotherapy	37	11.9
	Maintenance treatment	77	24.8
	Follow up	25	8.0

ECOG scoring criteria: (0: completely normal mobility; 1: able to walk freely and engage in light physical activities; 2: able to walk freely and take care of themselves; 3: only partially able to take care of themselves; 4: completely unable to take care of themselves; 5: death).

### 4.3 Correlation analysis

After the normality test, the scores for illness perception, social support, level of hope, and FOP were approximately normally distributed. Therefore, Pearson’s correlation was used to analyze the variables. **Table 2** presents the mean, standard deviation, and correlation between the variables. The results showed that the mean score of powerlessness in lymphoma patients was 37.85, with a standard deviation of 7.28. Hypothesis 1 was confirmed by a positive correlation between illness perception and FOP (r = 0.554, *P* < 0.01). The correlation between illness perception and social support was negative (r = -0.268, *P* < 0.01), as well as the correlation between illness perception and hope level (r = -0.489, *P* < 0.01). Social support was positively correlated with the level of hope (r = 0.493, P < 0.01) and negatively correlated with FOP (r = -0.483, *P* < 0.01). Additionally, the level of hope was negatively correlated with FOP (r = -0.545, *P* < 0.01).

**Table 2:** Descriptive statistics and correlation analysis of the variables (n = 311).

Variable	Mean ± SD	Disease perception	Social support	Hope level	FOP
Disease perception	47.07 ± 9.24	1			
Social support	34.7 ± 5.68	-0.268^**^	1		
Hope level	34.8 ± 5.46	-0.489^**^	0.493^**^	1	
FOP	37.85 ± 7.28	0.554^**^	-0.483^**^	-0.545^**^	1

**P < 0.01.

### 4.4 Analysis of intermediation effects

**Figure 2:**
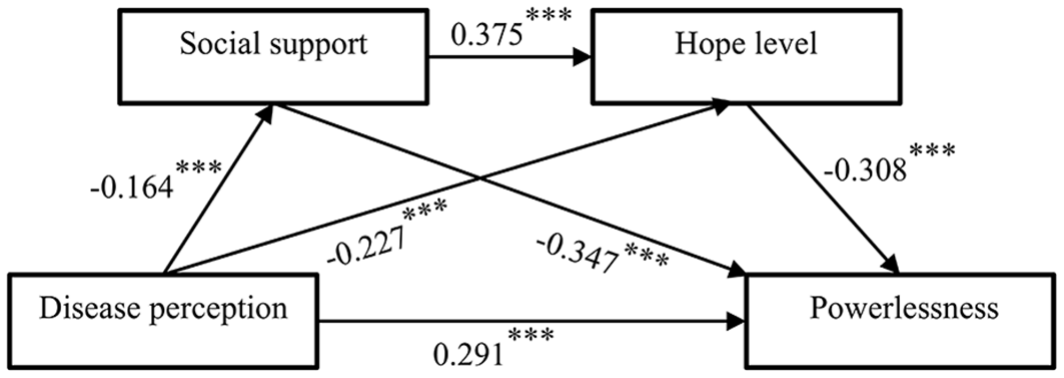
Chain-mediated model for lymphoma patients. (*** p < 0.001).

Using Model 6 in the SPSS27.0 process 4.1 plug-in prepared by Hayes, the results of the regression analysis showed (see **Table 3**, **Figure 2**) that the perception of illness in lymphoma patients significantly and positively predicted the FOP and had a negative predictive effect on the level of social support and hope; social support had a significant and positive predictive effect on the level of hope; social support and the level of hope had a negative predictive effect on the FOP.

**Table 3:** Regression analysis of the chain-mediated model of disease perception and powerlessness in lymphoma patients (n = 311).

Outcome variable	Predictor variable	R	R^2^	F	β	t
Social support	Disease perception	0.268	0.072	23.83 ^***^	-0.164	-4.882 ^***^
Hope level	Disease perception	0.617	0.381	94.802^***^	-0.227	-8.272 ^***^
	Social support				0.375	8.393 ^***^
FOP	Disease perception	0.679	0.461	87.494 ^***^	0.291	7.673 ^***^
	Social support				-0.347	-5.61 ^***^
	Hope level				-0.308	-4.335 ^***^

***P<0.001.

**Table 4** shows the results of the bootstrap mediation effect test. The study shows that: social support plays a partial mediation effect between the perception of disease and FOP, the effective value is 0.057, accounting for 13.04% of the total effect value, hypothesis 2 is confirmed; secondly, the level of hope also plays a partial mediation effect between the perception of disease and the FOP, the effective value is 0.07, accounting for 16.02% of the total effect value of 16.02%, and Hypothesis 3 was confirmed; in addition, the level of social support and hope played a partial mediating effect between perception of illness and FOP, with a valid value of 0.019, accounting for 4.35% of the total effect value, and Hypothesis 4 was also confirmed.

**Table 4:** Analysis of the mediating effect between perception of disease and the FOP in lymphoma patients (n = 311).

Trails	Effect value (β)	Percentage	Boot standard error	95% confidence interval		Significance
				Lower limit	Limit	
Aggregate effect	0.437	100.00%	0.037	0.363	0.51	√
Direct effect	0.291	66.59%	0.038	0.216	0.365	√
Total indirect effect	0.146	33.41%	0.026	0.096	0.198	√
Disease perception → social support → FOP	0.057	13.04%	0.016	0.029	0.091	√
Disease perception → level of hope → FOP	0.070	16.02%	0.019	0.034	0.107	√
Disease perception → social support → level of hope → FOP	0.019	4.35%	0.006	0.008	0.033	√

In summary, the results of this study validate the initially proposed hypothetical model (see **Figure 2**).

## 5 Discussion

We aimed to examine the relationship between illness perception and FOP in lymphoma patients, focusing on the mediating roles of social support and hope.

### 5.1 Relationship between illness perception and the FOP in lymphoma patients

In recent years, the FOP has gradually received attention from scholars; however, to date, no relevant studies have been reported in patients with lymphoma. In the current research, the average powerlessness score among patients with lymphoma was found to be 37.85. According to the Adult Powerlessness Scale, a score ranging from 37 to 48 is classified as severe, indicating that this patient group requires immediate attention regarding their feelings of powerlessness. One possible explanation for this finding is that individuals with lymphoma frequently encounter significant psychological challenges related to their condition and its treatment, which may include anxiety, depression, cognitive impairments, and difficulties with social adaptation. Marte et al. reported that patients with lymphoma often experience elevated psychological distress. However, these individuals frequently have limited access to mental health services. Furthermore, it has been noted that FOP can arise when physical, psychological, and social stressors are not effectively managed and persist over time. Our study also found a significant positive correlation between illness perception and powerlessness, which is consistent with our previously proposed hypothesis. The reason for this finding may be that the treatment of lymphoma typically involves multiple rounds of radiotherapy and/or chemotherapy, which could contribute to a significant impact on the psychological well-being of patients, potentially explaining the changes in their psychological state. During long-term treatment, patients not only need to endure physical symptoms and financial pressure but also face many psychological challenges (67). Specifically, the patients’ FOP may be closely related to their level of knowledge and understanding of the disease. If a patient’s cognitive appraisal of the disease is more negative, they feel unsure of the progress of the disease and the effectiveness of the treatment and at the same time question their ability to face the disease, thus generating a FOP. In contrast, patients with more positive perceptions of the disease are better able to evaluate their situation positively and take effective countermeasures to reduce their FOP. The results of the study suggest that the perception of illness plays an important role for patients and that medical practitioners should pay more attention to patients’ perceptions of illness to reduce their feelings of helplessness and despair.

### 5.2 Social support mediates the relationship between perceived illness and FOP

The results of the study showed that lymphoma patients with higher levels of social support had lower perceptions of powerlessness, which is consistent with previous studies (19, 68, 69). The findings also showed that social support partially mediated the relationship between illness perception and FOP, suggesting that social support may influence the relationship between how individuals perceive illness and feel powerless. This mediating role reveals the important influence of social support on an individual’s mental health. Specifically, the lack of adequate social support may increase individuals’ FOP when they perceive the severity of the disease. The reason for this analysis may be that when lymphoma patients face the challenges of the disease, their self-perceived social support can provide emotional, informational, or material comfort or assistance, which can help to reduce their psychological stress and enhance their sense of psychological security and self-efficacy, thereby reducing their FOP.

### 5.3 Levels of hope mediate the relationship between perceptions of illness and the FOP

The results of the present study indicate that patients with higher levels of hope perceive relatively lower FOP in the face of illness, a finding consistent with our previous hypothesis. In addition, it was further found that hope level partially mediated the relationship between illness perception and FOP, suggesting that the process of perceiving illness in individuals may influence the development of their FOP by altering their level of hope. Elevating an individual’s sense of hope may serve as a psychological intervention to help patients maintain a more positive mindset in the face of illness and reduce the occurrence of FOP. The reason for this may be analyzed as hope is a psychosocial force that gives patients the strength to live with a positive attitude in the face of great loss or extreme hardship (70). Particularly for lymphoma patients, their perceptions of the disease are influenced by the level of hope; the higher the level of hope, the more likely patients are to maintain a positive attitude towards life and have optimistic expectations for the future (16).This positive mindset motivates them to be able to cope with the disease in a more positive way, which in turn helps to enhance their self-efficacy and further reduces FOP.

### 5.4 Mechanisms of illness perception and FOP in lymphoma patients

In the present study, we found that social support was positively correlated with hope levels, which is consistent with previous studies (56–58). In addition, we found that social support and hope level play a mediating role between illness perception and FOP. Social support and hope level are not only independent mediating variables, they interact with each other to form a chain mediating effect. In terms of the mechanism of action, the results of this study are consistent with the CSM model. This result may suggest that illness perception may indirectly affect patients’ FOP by influencing social support and hope levels. This finding may suggest that for treating patients with chronic diseases such as lymphoma, it is important to focus not only on enhancing patients’ social support, but also on increasing their level of hope. These may become important tools for improving treatment outcomes and reducing FOP. By testing the chain mediation model, we gained a deeper understanding of the relationship between illness perception and FOP. Our findings may suggest that social support and level of hope are important psychological factors influencing FOP in lymphoma patients, which provides a clear direction for future nursing interventions. Caregivers can help patients face their disease more positively by increasing their social support and enhancing their sense of hope, especially in the early stages of the disease or at critical moments in the course of the disease, which in turn improves their mental health and quality of life. Health education and psychological counselling are used to help patients develop a positive perception of their illness and improve their ability to cope with it; communication and mutual support among patients can also be promoted by organizing their participation in support groups and psychological intervention programs, so that they can work together to cope with the challenges of their illness and improve their overall quality of life.

In summary, the results of this study show that illness perception not only directly affects patients’ FOP but also affects it through three indirect pathways. Specifically, illness perception can affect powerlessness through social support, i.e., illness perception → social support → powerlessness; in addition, illness perception can also affect powerlessness by influencing the level of hope, forming a pathway of illness perception → level of hope → powerlessness; lastly, illness perception can indirectly affect powerlessness through the joint effect of social support and level of hope, with the complete pathway of illness perception → social support → level of hope → powerlessness; and the complete pathway of illness perception → level of hope → powerlessness. Finally, illness perception can indirectly affect powerlessness through the combined effect of social support and hope levels, forming the complete path of illness perception: social support, hope level, and powerlessness. These findings suggest that social support and hope play important mediating roles in the powerlessness of lymphoma patients. Therefore, in the future, enhancing patients’ perceptions of social support and increasing their level of hope may be effective in reducing their FOP and thus improve their quality of life.

## 6 Implications for clinical practice

This study sheds new light on the emotional and psychological experiences of patients with lymphoma by examining the connections between illness perception, social support, hopefulness, and feelings of helplessness using a mediated effects model. The outcomes not only confirm the CSM theoretical framework but also provide vital groundwork for enhancing the quality of life of patients with lymphoma. In addition, our findings offer practical insights into clinical practice. We suggest that caregivers work to strengthen patients’ social support systems and elevate their hope levels, as this may effectively alleviate patients’ FOP and improve their overall well-being and quality of life.

## 7 Limitations and future research directions

First, the sample in this study was region-specific; therefore, the generalizability of these findings may be somewhat limited by the characteristics of the sample, the treatment methods employed, and the duration of follow-up. To enhance the validity and generalizability of the conclusions drawn from this study, future research should aim to increase the sample size, prolong the follow-up period, and validate the findings across diverse regions and populations. Second, in this study, we failed to control for demographic variables, which may have had some confounding effect on the results. Future studies should consider including controls for demographic variables to further validate the stability and broad applicability of the findings. Furthermore, the bias of the cross-sectional design and potential confounding factors such as comorbidities, lymphoma type, stage, and previous treatment. Time constraints necessitated our research to capture a snapshot of a situation at a single point in time, it may not capture the full picture of the phenomenon being studied, this methodology limits the ability to fully trace the causal chain of events. Conducting longitudinal studies monitoring changes over extended periods would provide valuable insights. Despite these limitations, our study offers important insights into the factors contributing to the FOP among lymphoma patients and suggests potential avenues for future research and nursing practice.

## 8 Conclusion

In summary, we found that illness perception and FOP were positively correlated in patients with lymphoma. Furthermore, the levels of social support and hope not only serve as independent mediators between illness perception and FOP, but also exhibit a chain mediating effect. This underscores the importance of illness perception, social support, and hope in psychological interventions for patients with lymphoma and provides a theoretical foundation and guidance for future caregivers to develop effective interventions aimed at reducing FOP.

## Data Availability

The original contributions presented in the study are included in the article/supplementary material. Further inquiries can be directed to the corresponding author/s.
